# Using intrahost single nucleotide variant data to predict SARS-CoV-2 detection cycle threshold values

**DOI:** 10.1371/journal.pone.0312686

**Published:** 2024-10-30

**Authors:** Lea Duesterwald, Marcus Nguyen, Paul Christensen, S. Wesley Long, Randall J. Olsen, James M. Musser, James J. Davis

**Affiliations:** 1 College of Engineering, Cornell University, Ithaca, NY, United States of America; 2 Northwestern-Argonne Institute for Science and Engineering, Evanston, IL, United States of America; 3 Data Science and Learning Division, Argonne National Laboratory, Lemont, IL, United States of America; 4 Consortium for Advanced Science and Engineering, University of Chicago, Chicago, IL, United States of America; 5 Laboratory of Human Molecular and Translational Human Infectious Diseases, Center for Infectious Diseases, Houston Methodist Research Institute and Department of Pathology and Genomic Medicine, Houston Methodist Hospital, Houston, TX, United States of America; 6 Department of Pathology and Laboratory Medicine, Weill Cornell Medical College, New York City, NY, United States of America; Centers for Disease Control and Prevention, UNITED STATES OF AMERICA

## Abstract

Over the last four years, each successive wave of the COVID-19 pandemic has been caused by variants with mutations that improve the transmissibility of the virus. Despite this, we still lack tools for predicting clinically important features of the virus. In this study, we show that it is possible to predict the PCR cycle threshold (Ct) values from clinical detection assays using sequence data. Ct values often correspond with patient viral load and the epidemiological trajectory of the pandemic. Using a collection of 36,335 high quality genomes, we built models from SARS-CoV-2 intrahost single nucleotide variant (iSNV) data, computing XGBoost models from the frequencies of A, T, G, C, insertions, and deletions at each position relative to the Wuhan-Hu-1 reference genome. Our best model had an R^2^ of 0.604 [0.593–0.616, 95% confidence interval] and a Root Mean Square Error (RMSE) of 5.247 [5.156–5.337], demonstrating modest predictive power. Overall, we show that the results are stable relative to an external holdout set of genomes selected from SRA and are robust to patient status and the detection instruments that were used. This study highlights the importance of developing modeling strategies that can be applied to publicly available genome sequence data for use in disease prevention and control.

## Introduction

The COVID-19 pandemic, caused by severe acute respiratory syndrome coronavirus 2 (SARS-CoV-2), has had an extreme public health impact over the last four years. Since its emergence, it has caused over 773 million confirmed cases and over 7 million deaths worldwide [1]. The SARS-CoV-2 virus has evolved over time to become more transmissible, resulting in new variants of concern (VOCs) and causing successive waves of infection [2, 3]. This sequential and ongoing emergence of VOCs, such as those observed in late 2020 with Alpha, followed by Delta, Omicron, and the subsequent descendants of Omicron [4], present a substantial public health threat. Despite this, the bioinformatic identification of new VOCs remains challenging and usually occurs only after there is community transmission of the new variant, hampering efforts to control viral spread [5, 6].

Clinical testing has played an important role in the pandemic response, enabling early identification and intervention. Real-time reverse-transcription polymerase chain reaction (RT-PCR) tests are the gold standard molecular diagnostic for detecting SARS-CoV-2 [7]. The viral RNA in a patient sample, most commonly collected via a nasal swab, is converted to DNA by reverse transcription, and then amplified by PCR until the resulting SARS-CoV-2 cDNA is detectable. The cycle threshold (Ct) value of a positive SARS-CoV-2 test is the number of rounds of PCR amplification that are necessary for the amplified sequence to reach the point where it becomes detectable by the clinical detection instrument. Although there are many factors that can influence the Ct value observed in a clinical test including time since infection, sample preparation and quality, and differences in detection reactions and instruments [8], Ct values tend to be inversely correlated with viral load, providing a useful approximation of the viral RNA in a patient sample [9–11].

Ct values can also serve as a valuable source of epidemiological data. For example, Ct values for cross-sectional samples collected from patient populations over a given time period are often indicative of the state of the epidemic, with lower average Ct values indicating a growing pandemic [12–15]. Similar studies have also found that cross-sectional trends in Ct values can act as indicators of the future trajectory of the pandemic [16, 17]. At the patient level, Ct values have been shown to provide a good estimate of how long a patient will remain contagious [10, 18, 19], and several studies have shown that lower Ct values (i.e., higher viral loads) can be correlated with symptomatic infection, morbidity, and mortality [9, 14, 20, 21]. Higher viral loads and increased transmissibility have been reported for each successive VOC as it has emerged, including the Alpha, Delta, and Omicron variants [22–24].

Because VOCs significantly alter the course of the pandemic and threaten the efficacy of important countermeasures, including vaccines and the effectiveness monoclonal antibody treatments, developing tools for the early identification of variants with the potential for increased transmissibility is important. Such early identification could enable prompt clinical responses to greatly limit, or even prevent, the spread and impact of a new variant. Genomic surveillance studies have aggregated data from public repositories to develop models to identify mutations involved in transmissibility and identify notable variants with the potential to spread [6, 25–27]. Similarly, studies analyzing whole genome and individual protein sequences using statistical and machine learning methods have also been developed to predict disease severity [28, 29], vaccine targets [27, 30], transmissibility [26], and future variants of concern [31–33]. However, to date, these modeling strategies have had only a modest impact on public health response strategies.

Most of the genome sequencing that has been performed for SARS-CoV-2 clinical samples has been based on amplicon sequencing with reference-based assembly, where the sample is amplified using an established set of primers and the reads are aligned against the Wuhan-Hu-1 reference genome. When the reads from the sample are aligned against the reference genome, it is common to observe variation or intrahost single nucleotide variants (iSNVs), at any given column in the alignment. These minor variants go unobserved in the assembly because a consensus base is chosen for each position using a statistical model [34].

In previous work, we built machine learning models for predicting Ct values from assembled SARS-CoV-2 genome sequences [35]. Our best model, which was based on 29,000 genomes, had a modest predictive signal (R^2^ = 0.521). Previous studies have shown a relationship between iSNVs and Ct values, with higher Ct value samples having higher iSNV frequencies [36, 37]. In this study, we explore using iSNV frequencies for improving the prediction of SARS-CoV-2 Ct values.

## Results

### Models built from iSNV frequencies are predictive

**Fig 1:**
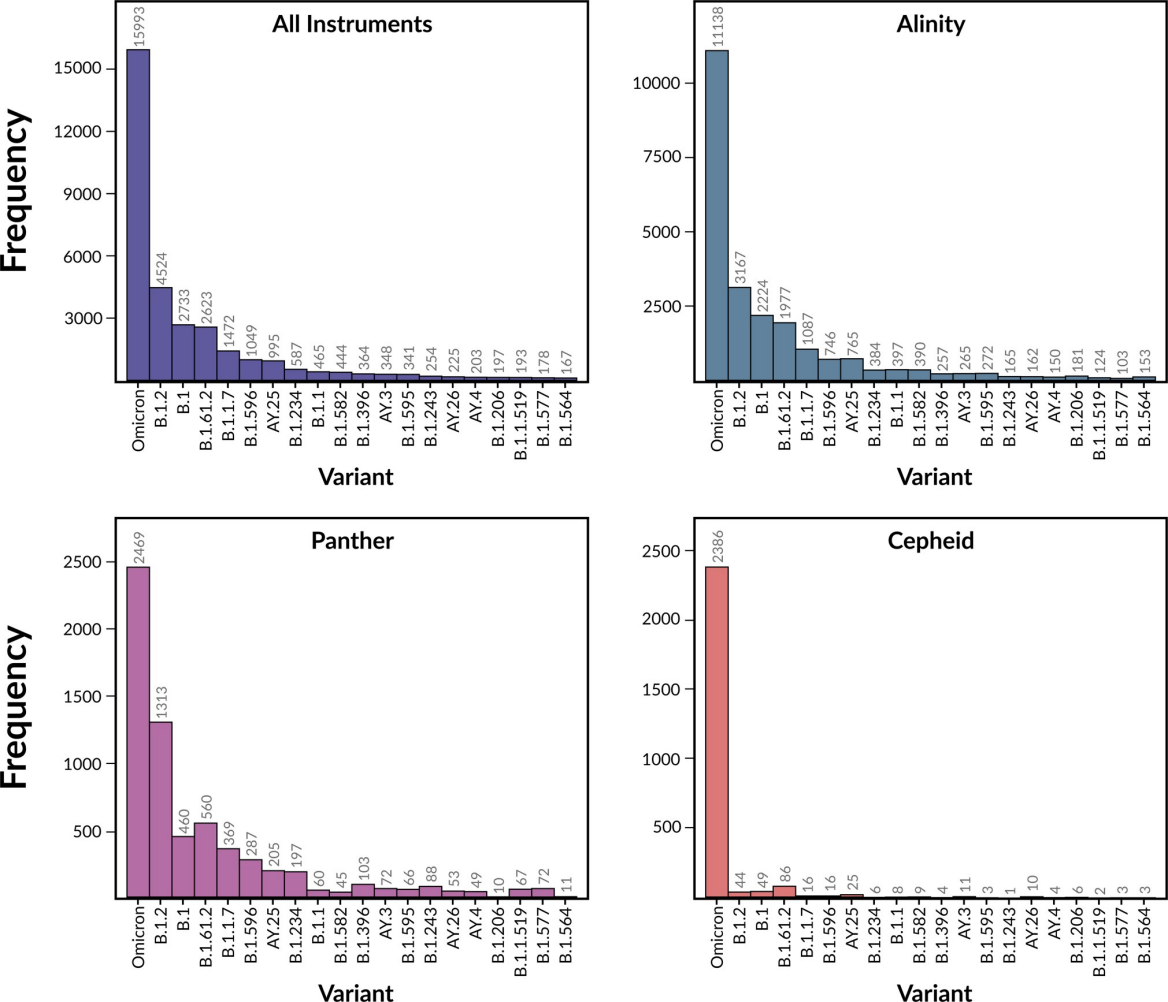
Histograms showing the distributions of the 20 most common variants in the dataset.

**Fig 2:**
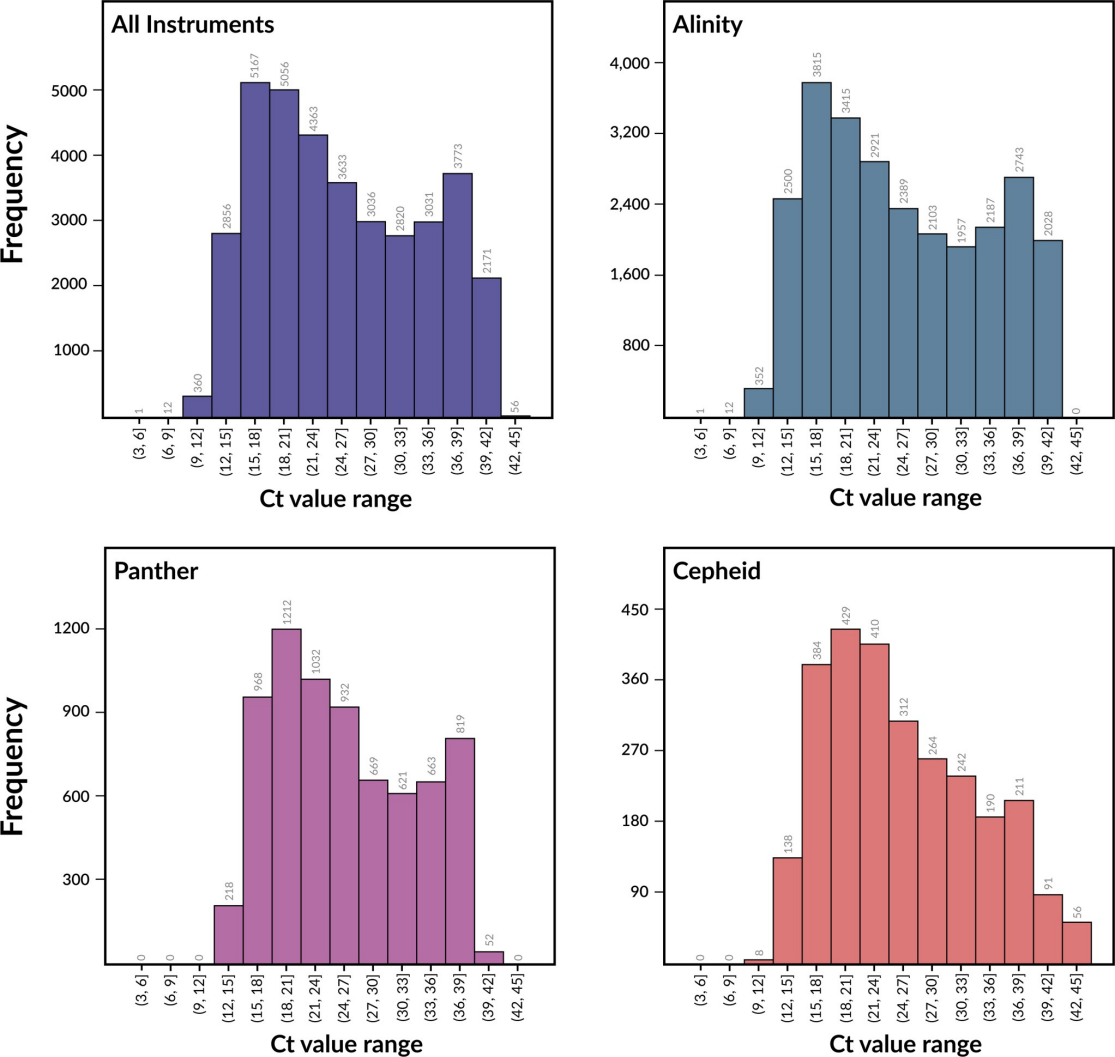
Histograms showing the distributions of Ct values in the dataset. Ct values were sorted into bins of 3 with an inclusive lower bound and exclusive upper bound.

A total of 36,335 SARS-CoV-2 clinical samples, collected over a period of 31 months from July 1, 2020, to March 21, 2023, were used in this study. All positive samples were identified on one of three clinical detection platforms including Alinity (26,414 samples), Panther (7,186 samples), and Cepheid (2,735 samples) (**Fig 1**). The corresponding high quality sequenced genomes contain 301 named variants of SARS-CoV-2 with 96 named variants occurring 10 or more times. The most common variants during the sampling period were Omicron and its sub-lineages, Delta and its sub-lineages, Alpha, and the related B.1 and B.1.2 variants from early in the pandemic (**Fig 1** and **S1 Table in S1 File**). The Ct values in the dataset ranged from 5.1 to 45.0 with a median of 24.3, and a standard deviation of 8.4 (**S1 Table in S1 File**). Samples from the three detection instruments had mean Ct values of 25.5–25.6 and median Ct values of 24.0–24.5 (**S2 Table in S1 File**). The distributions of Ct values over the set of samples are similar for Alinity and Panther. The Cepheid distribution differs slightly and has a smaller number of samples (**Fig 2**). We note that an upper Ct value cutoff of 45 is considered high by most laboratory standards. These samples are included in the analysis because they were considered to be clinically positive. In particular, the Cepheid system considers samples positive when the E and N2 genes are detected in less than 45 cycles [38]. Nevertheless, we control for potential effect of the Ct value range below.

**Fig 3:**
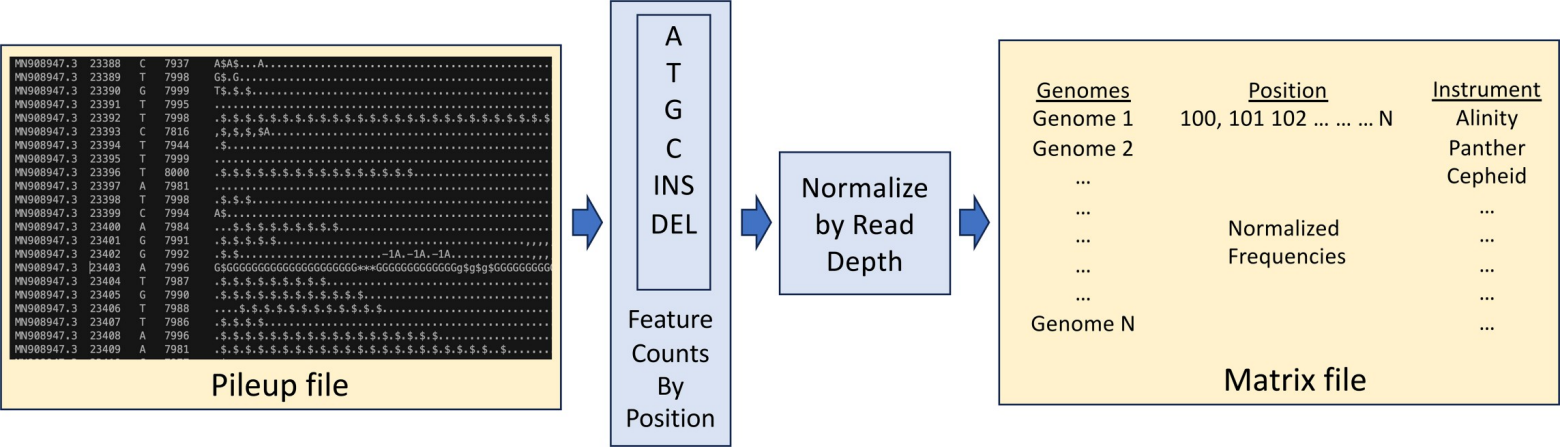
Schematic of the matrix generation for the XGBoost models. Pileup files were generated by aligning reads against the reference genome, and the iSNV frequencies of A,T,G,C, insertions, and deletions at each position were computed per genome and normalized with respect to read depth. Normalized iSNV values and the one-hot-encoded clinical detection instruments were used to create the matrix that was used to generate the XGBoost models.

The main objective of this study was to determine if models trained on the iSNV data encoded in the reads could be used to improve the prediction of Ct values. To do this, we constructed feature matrices from the pileup files [39], capturing the frequencies of A,T,G,C, insertions, and deletions at each position relative to the Wuhan-Hu-1 reference genome (**Fig 3**). Regression models were built using Extreme Gradient Boosting (XGBoost) [35]. The average R^2^ and root-mean-square error (RMSE) for the model built from all 36,335 genomes were 0.604 [0.593–0.616, 95% confidence interval] and 5.247 [5.156–5.337], respectively (**Table 1**), indicating that the models provide predictive signal, and a statistically significant improvement upon our previous model that was built using only the assembled genomes, which had an R^2^ score of 0.521 ± 0.010 and an RMSE of 5.7 ± 0.034 [35].

**Table 1:** Ct value prediction results for a model built using all detection instruments and separate models for each instrument.

Model	Genomes	R^2^	RMSE
All Instruments	36,335	0.604 [0.593–0.616]	5.247 [5.156–5.337]
Alinity only	26,414	0.606 [0.599–0.614]	5.482 [5.419–5.545]
Panther only	7,186	0.559 [0.530–0.588]	4.770 [4.616–4.925]
Cepheid only	2,735	0.566 [0.525–0.608]	5.187 [4.929–5.444]

### Models built from all detection instruments are robust

In order to assess how the model performance is influenced by each detection instrument and the inherent differences in detection targets and the reporting of cycle numbers or cycle thresholds, separate models were trained using the genomes corresponding to each clinical detection instrument. The model trained on the most common instrument in the dataset, Alinity with 26,414 genomes, had an R^2^ score of 0.606 [0.599–0.614] and an RMSE of 5.482 [5.419–5.545], and was nearly identical to the model trained with all instruments (**Table 1**). The models trained using data from only Panther and Cepheid samples demonstrated slightly poorer performances. The Panther-only model (7,186 genomes) had an R^2^ score of 0.559 [0.530–0.588] and an RMSE of 4.770 [4.616–4.925], and the Cepheid-only model (2,735 genomes) had an R^2^ score of 0.566 [0.525–0.608] and an RMSE of 5.187 [4.929–5.444]. The reduced accuracy of the Panther and Cepheid data sets is likely due to the smaller number of genomes in those data sets.

Similarly, we evaluated how well the all-instrument model performed using the test set data from each separate instrument. In these cases, none of the R^2^ values are significantly different from the models trained separately for each instrument (**Table 2**). Overall, these data show that combining all of the instruments into the same model does not negatively impact the predictions for each instrument in the test set.

**Table 2:** Results for the all-instrument model showing the R^2^ and RMSE values for each instrument in the test set averaged over each fold.

Instrument	Total Genomes	Fraction of Training Set	R^2^	RMSE
Alinity	26,414	0.727	0.615 [0.600–0.630]	5.377 [5.245–5.509]
Panther	7,186	0.198	0.567 [0.529–0.604]	4.748 [4.542–4.955]
Cepheid	2,735	0.075	0.565 [0.544–0.587]	5.202 [4.932–5.472]

### Models are robust to patient status

Because the data set almost entirely comprised of samples from patients with varying levels of disease burden and co-morbidities, and these sets are not balanced, we computed the R^2^ and RMSE scores for each category using the all-instrument model. Overall, outpatient samples have a slightly lower median Ct value of 25.0 compared with 26.4 for the inpatients. Among inpatients, the intermediate medical unit (IMU) and intensive care unit (ICU) samples have average Ct values of 27.0 and 26.6, respectively (**S3 Table in S1 File**). The R^2^ value for the outpatient set (0.615 [0.606–0.623]) was not significantly different than the inpatient set (0.578 [0.551–0.606]), and R^2^ for the IMU sets (R^2^ = 0.623 [0.496–0.751]) was also not significantly different than the ICU set (0.560 [0.519–0.602]). Although there are likely too few IMU samples (280) in the analysis do draw a conclusion, these results suggest that potential data imbalances due to outpatient, inpatient, and ICU status have a negligible impact on the model performance.

### Model accuracy across the range of Ct values

**Fig 4:**
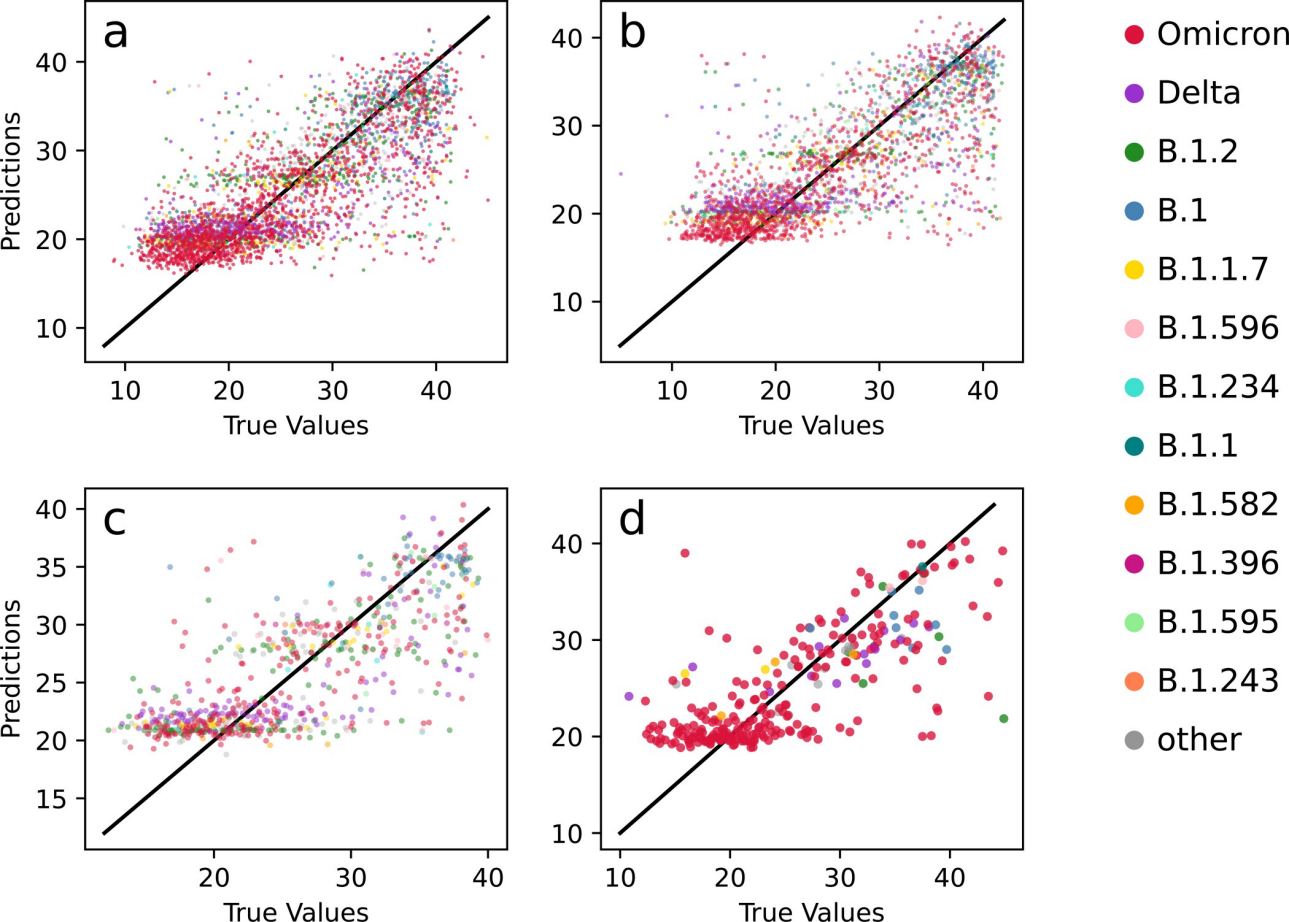
Scatterplots of predicted versus actual Ct values. Scatterplots were constructed for models trained and tested on the following instruments: **a)** All instruments, **b)** Alinity, **c)** Panther, **d)** Cepheid. Points are colored by variant with samples of the 10 most frequently occurring variants colored via the key shown in the right, and samples of other variants colored gray. The line y = x is shown across the center diagonal of the figure for reference. Data are from a single fold.

In order to understand how well the model worked over the range of Ct values, we plotted the predicted versus actual values for the all-instrument model for a single fold of the 10-fold cross validation (**Fig 4**). In general, the plot of predicted versus actual Ct values forms a diagonal distribution, indicating accurate predictions, and this result is consistent across each detection instrument. Although there are incorrect predictions scattered throughout the plot, the bulk of the inaccuracies occur in samples with Ct values less than 20, which is consistent with higher viral load samples having fewer detectable iSNVs [36, 37].

**Fig 5:**
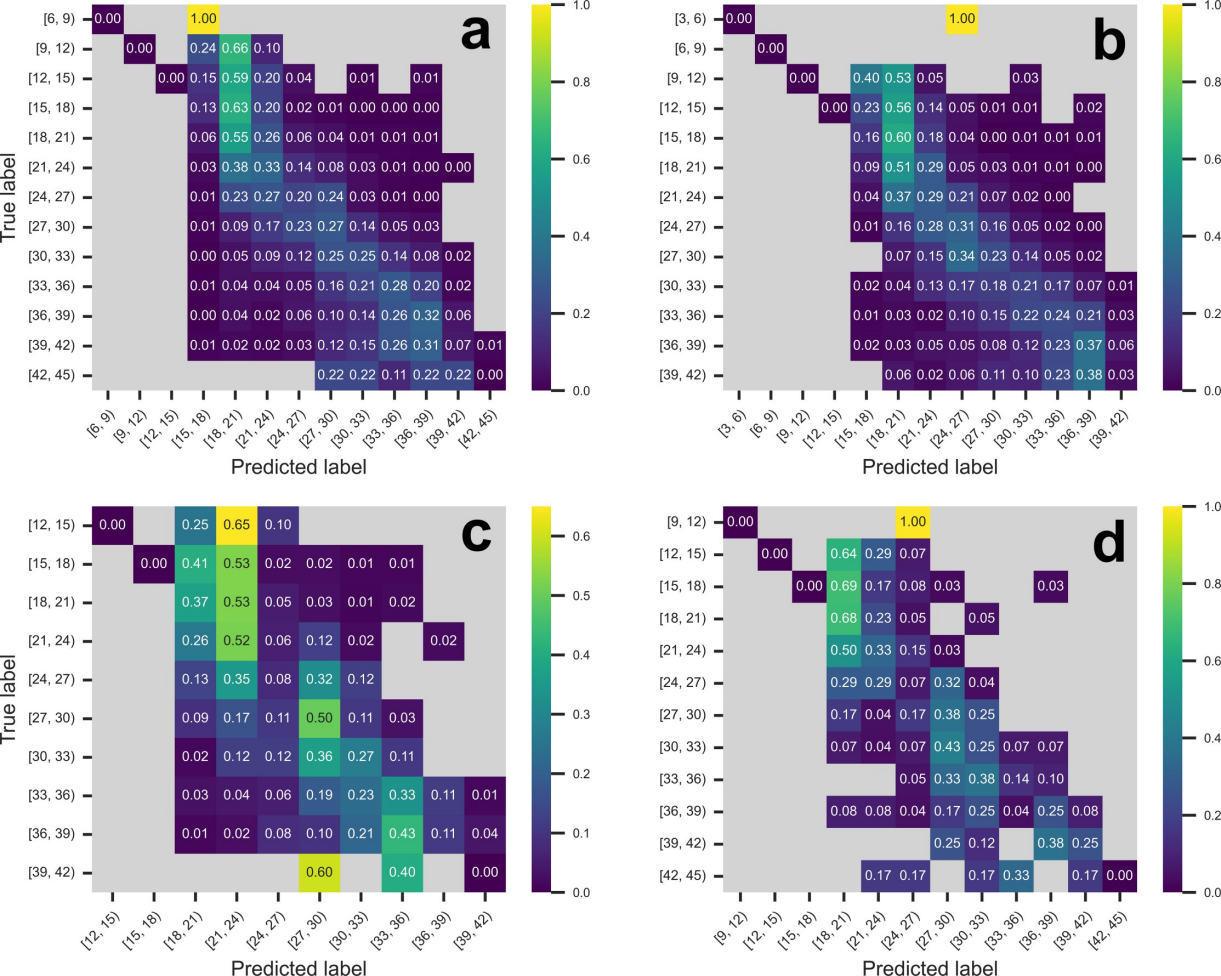
Confusion matricies for models by instrument using a single fold. Model predictions were binned into Ct value ranges of 3 cycles with an inclusive lower bound and exclusive upper bound. Coloring and values in each cell represent the fraction of the actual Ct values predicted in the given interval. Empty cells with no predictions or actual values in that range are gray. Confusion matrices were constructed for models trained and tested on the following instruments: **a)** All instruments, **b)** Alinity-only, **c)** Panther-only, and **d)** Cepheid-only.

To understand the bounds of the accuracy of the all-instrument model, we binned predicted and actual Ct values into bins of size 3 cycles, plotting the results in a confusion matrix heatmap (**Fig 5**). Like the scatterplot, the predictions form a mostly diagonal pattern, corresponding to the accurate predictions. For both the all-instrument model and the individual instrument models, we observe the most errors in samples with Ct values between 6–18.

In our clinical sampling protocol, samples were considered to be positive if they had Ct values ≤45. Previous studies have considered Ct values above 35 as being weakly positive [40, 41], and a high profile early study classified PCR positive samples with Ct values above 38 as being negative [29]. Since the samples with high Ct values could be a source of error, we built a series of separate models where we excluded all samples with Ct values above a given threshold (**Table 3**). Overall, removing all samples with Ct values greater than 40 does not result in models with significantly different R^2^ or RMSE values relative to the model built from all samples and all Ct values, or the model built from a matched number of samples selected randomly. As the threshold goes below a Ct value of 36, we begin to see models with R^2^ values going below 0.5, although these more restrictive models still have some predictive power. Models that were built from the same number of genomes, where the samples were removed randomly to create a matched sample size, showed no significant difference in R^2^ or RMSE. These results indicate that the model requires a range of Ct values, but that the high Ct values, particularly those greater than 38, are not the main reason for the model’s predictive power.

**Table 3:** R^2^ and RMSE scores for models built from samples with successively lower Ct values, and the corresponding results from models built with the same number of samples removed randomly.

		Models with high Ct value samples removed		Models with a matching number of samples randomly removed	
Ct value cutoff	Genomes	R^2^	RMSE	R^2^	RMSE
< 40	35,099	0.586 [0.568–0.603]	5.149 [5.030–5.268]	0.588 [0.582–0.595]	5.369 [5.307–5.432]
< 38	32,629	0.557 [0.539–0.575]	4.861 [4.779–4.942]	0.601 [0.595–0.606]	5.283 [5.262–5.305]
< 36	30,305	0.517 [0.503–0.531]	4.580 [4.501–4.659]	0.600 [0.593–0.607]	5.276 [5.251–5.301]
< 34	28,244	0.459 [0.445–0.472]	4.375 [4.293–4.457]	0.600 [0.584–0.616]	5.302 [5.193–5.410]
< 32	26,337	0.408 [0.389–0.427]	4.092 [3.997–4.187]	0.583 [0.563–0.603]	5.428 [5.281–5.575]
< 30	24,445	0.341 [0.330–0.352]	3.886 [3.839–3.934]	0.600 [0.586–0.614]	5.299 [5.212–5.386]

Although the R^2^ value of the all-instrument model indicates that the model has predictive value, it is difficult to understand this in terms of accuracy. To approximate the overall accuracy of the model, we computed the accuracy of the model within a range of Ct values (**Table 4**). Overall, the model approaches 50% accuracy within a window of ±3 cycles, and 80% accuracy within ±6 cycles. We also selected an external holdout set of 1,795 whole genome SARS-CoV-2 sequences from SRA (**S4 Table in S1 File**), for which the Ct values had been reported using primers for the N and ORF1ab genes. Surprisingly, the model performs slightly better over this holdout set (**Table 4**), although we note that the sampling time frame, between February and June of 2021, is much shorter.

**Table 4:** The accuracy of the all-instrument model for predicting Ct values within a window size varying from 1–6. Data are shown for the test set of the all-instrument model as well as a holdout set of 1,795 SARS-CoV-2 genomes from SRA.

Ct Value Window Size	Test Set (Internal)	Holdout Set (External)	
	All instruments (E, Orf1ab, N, N2, RdRp genes)	N gene	ORF1ab
±1	0.174 [0.170–0.177]	0.223 [0.216–0.230]	0.213 [0.196–0.229]
±2	0.341 [0.340–0.343]	0.423 [0.405–0.440]	0.408 [0.388–0.427]
±3	0.490 [0.484–0.495]	0.581 [0.554–0.608]	0.578 [0.566–0.590]
±4	0.611 [0.609–0.613]	0.705 [0.676–0.733]	0.719 [0.710–0.728]
±5	0.714 [0.704–0.723]	0.803 [0.761–0.846]	0.826 [0.811–0.840]
±6	0.794 [0.786–0.802]	0.877 [0.841–0.913]	0.902 [0.879–0.924]

### Feature importance

**Fig 6:**
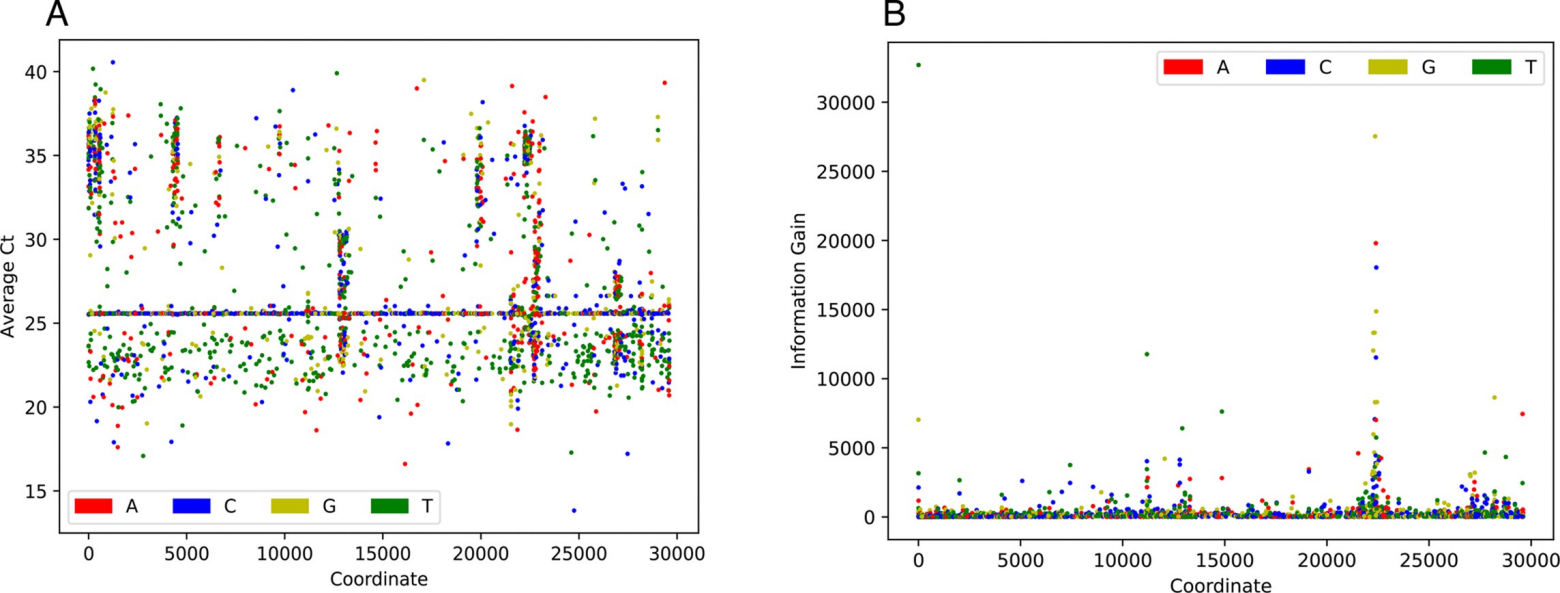
Dot plot depicting the average Ct value (A) and XGBoost feature information gain (B) for each position and character used by the all-instrument model. Each base at a given position is colored according to the key. Genomic positions correspond to the SARS-CoV-2 Wuhan-Hu-1 reference genome. For each position, only genomes where ≥40% of the characters in the column corresponded to a given nucleotide were used to generate the average gain in order to reduce noise in the image. Additionally, only statistically significant bases are included, significance was computed based on the 95% confidence interval of the average Ct value of genomes with a given base and those without. No INDEL features met this significance requirement. The spike protein corresponds to genomic coordinates 21563–25384.

In order to understand the genomic regions that contain iSNVs that are potentially linked to differences in Ct values, we plotted the average Ct values for the genomes encoding either A, T, G, C, insertions, or deletions at a given position (**Fig 6A**). There are several regions in the Wuhan-Hu-1 reference genome where certain bases correspond with differences in Ct values, including a large cluster within the gene encoding the spike protein.

To see how iSNVs potentially influence the models, we plotted the XGBoost feature importance as gain (**Fig 6B**) and weight (**S1 Fig**). Overall, both plots show a concentration of highly ranked features in the spike gene, although there are also other areas with highly ranked features elsewhere. This suggests that iSNVs in the spike gene have an impact on the Ct values and are important for the predictions made by the models. While the features with the highest importance in spike cluster near amino acid positions 280–330 it is important to note that the XGBoost models are greedy and may not need to choose all of the positions with iSNVS that potentially influence the Ct values.

### Predicting Ct values for a new VOC

**Fig 7:**
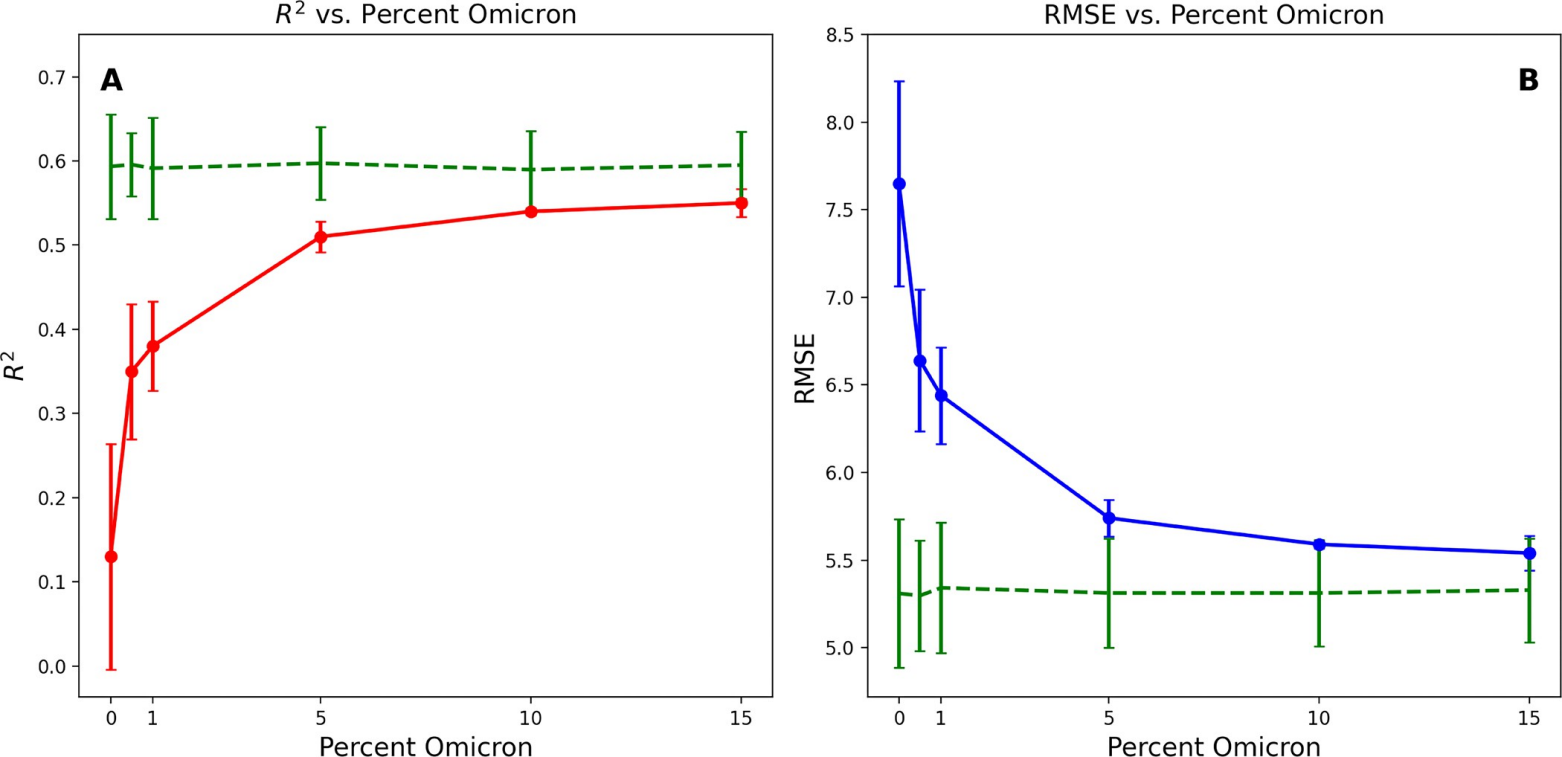
The A) R^2^ (red line), and B) RMSE (blue line) for a holdout set of Omicron genomes using models trained on increasing percentages of Omicron genomes in the training set. The green dashed line depicts the R^2^ and RMSE for the training set, which contains no Omicron genomes.

Due to the potential importance of being able identify new VOCs, we evaluated the ability of a model to predict the Ct values of a newly emerging variant. To do this, we first trained an all-instrument model as before, but removed the Omicron genomes from the training set to simulate a model that was created before the emergence of Omicron. We then evaluated this model on a test set containing only Omicron genomes. The model trained without Omicron genomes starts out with a low R^2^ of 0.131 [0.064–0.198] but rapidly increases to 0.547 [0.539–0.555] as the Omicron reaches 15% of the samples (**Fig 7**) and begins to approach the average R^2^ of the model that was trained from non-Omicron samples, 0.594 [0.570–0.594]. An analogous trend is seen in the RMSE, which decreases as Omicron genomes are added. These results indicate that some Omicron data is required for the models to learn the Ct values for Omicron samples. This is perhaps unsurprising given the remarkable genomic differences between Omicron and previous VOCs, and is consistent with our previous observations building models from assembled genome sequences [35].

## Discussion

In this work, we built models using iSNVs from short read sequence data in order to predict the cycle threshold values for clinical SARS-CoV-2 samples. Pileup files, which are alignments of the raw reads of a clinical sample against a reference genome, were used to compute the iSNVs. This resulted in models with R^2^ and RMSE values that were significantly better than what we had shown previously for models built from assembled whole genome sequences [35]. Furthermore, by using the iSNVs we observed a much clearer pattern in the feature importances, which implicated the region of the genome encoding the spike protein. However, we note that the predictive power of this model is still modest and requires improvement before an approach like this can be applied in a practical setting.

Predicting Ct values is a challenging modeling problem because of the complexities of the data and patient status. A patient’s viral load would be expected to increase after they contract the disease, and then eventually go down as they clear the virus, so time since infection is an important factor that is not captured by the sequence data alone and was not modeled in this study. Sampling and handling errors, intrinsic error in the detection instruments, and differences the detection primers used in each assay, are also expected to add noise to the models. These effects can also be difficult to capture when looking at only at the raw sequence data. Although we chose not to do so in this study, correcting for an error rate of up to 2 cycles would be supported by the current literature [42], and could be expected to be even higher using different detection protocols [43]. Indeed, even the two different gene targets in the external holdout set in **S4 Table in S1 File** had an average CT value difference of almost 1 cycle for the same clinical sample.

One particular technical caveat that we happened upon in this study that is worth noting, is that models built from the pileup files are sensitive to read depth. Indeed, without normalizing for read depth, we observed R^2^ values as high as 0.73. Upon further investigation, we found that the model was learning the differences in read depth at certain positions that naturally occurred throughout the pandemic due to the declining effectiveness of old primers, and the altered characteristics of updated primers.

Having the ability to identify new variants early, before there is community transmission, is necessary for controlling the spread of the virus as well as for taking measures to predict new variants of concern and to predict vaccine effectiveness. This study demonstrates that it is possible to use SARS-CoV-2 genome sequence data to predict Ct values, which correspond with viral load. However, the modeling strategy still requires considerable improvement before it can be used to generate actionable predictions. By successively adding Omicron sequences to a training set lacking Omicron, we showed that approximately 15% of the training set needed to be comprised of Omicron sequences before the models could predict Ct values for the Omicron genomes with a similar R^2^ to the other variants in the training set. This indicates that although the model learns well, more research is required if we wish to predict the Ct value from the sequence of a novel variant. Adding additional information to the models relating to the effects of amino acid changes to epitope sites, or protein structures could help. Advances in artificial intelligence techniques, particularly in large language modeling, may also offer a means of improvement.

During the pandemic, sequence data proved to be an integral part of monitoring the outbreak and making subsequent public health decisions, despite the potential delays in depositing the data and the incompleteness of the associated metadata [44]. This study highlights the value of being able to predict clinical characteristics of SARS-CoV-2 variants from short read data for epidemiology and infection control. At the time of writing, there are over seven million SARS-CoV-2 sequences in the NCBI Sequence Read Archive, but despite this trove of data, we still lack the models that would be necessary to inform a proactive outbreak response. Continued effort in the development of modeling strategies that leverage public data, as well as the identification of valuable metadata that will improve predictions and thus should be deposited, will help future infection control efforts.

## Materials and methods

### Data collection

A total of 36,335 SARS-CoV-2 genome sequences were used in this study. The sequences were collected across the Houston Methodist Hospital system and from institutions using the Houston Methodists diagnostic laboratory services between July 1, 2020 and March 21, 2023. All samples were collected from nasopharyngeal swabs immersed in universal transport media. All methods were performed in accordance with the relevant guidelines and regulations, and all experimental protocols were approved by the Houston Methodist Research Institute (Pro00005073:1, Houston Methodist Research Institute Institutional Review Board). A waiver of consent for retrospective studies was granted by the Houston Methodist Research Institute IRB. All sample dates are provided in **S1 Table in S1 File**. All patient data used in this study were deidentified prior to analysis. Positive clinical samples and Ct values were generated on multiple clinical detection platforms. The dataset in this study is comprised of Ct values from three detection systems: the Abbott Alinity *m* SARS-CoV-2 AMP kit (Abbot Molecular Inc., Des Planes, IL, USA), the SARS-CoV-2 Assay using the Hologic Panther Fusion System (Hologic, Marlborough, MA), and the Xpert Xpress SARS-CoV-2 test using Cepheid GeneXpert Infinity or Cepheid GeneXpert Xpress IV instruments (Cepheid, Sunnyvale, CA). The Alinity system amplifies regions of the N and RdRp genes [45], the Panther system amplifies the Orf1ab gene [46], and the Cepheid system amplifies the N2 and E genes [38]. We note that that the Alinity *m* system returns a qualitative cycle number CN value, rather than a true CT value. This has been shown to have strong concordance with CT values from other tests [42], and is referred to as a CT value herein for simplicity.

Upon clinical detection, samples were amplified for sequencing using either the ARTIC V3, V4, or V4.1 primers (V4 for collection dates after July 28, 2021, and V4.1 for collection dates after Jan 5, 2022) (https://artic.network/ncov-2019) using methods described previously [47–49] (**S1 Table in S1 File**). All of the genomes were sequenced using an Illumina NovaSeq 6000 instrument (Illumina, San Diego, California, USA).

### Genome quality filtering

In order to assess genome quality, all genomes were assembled with the BV-BRC SARS-CoV-2 assembly service [50] (https://www.bv-brc.org/app/ComprehensiveSARS2Analysis), which performs a reference-based assembly against the Wuhan-Hu-1 reference genome (GenBank ID: MN908947.3). The pipeline uses minimap version 2.143 [51] for aligning reads against the reference and iVar version 1.2.2 for primer trimming and SNP calling [34]. Default parameters were used in all cases except that the maximum read depth in mpileup [39] was limited to 8,000, and the minimum read depth was set to 3 in iVar. All variants were identified using Pangolin version 4.0.3 (https://cov-lineages.org/resources/pangolin.html).

Each genome was sampled from a unique patient, and the set of genomes used in the study was down selected from a larger set of over 100,000 genomes in order reduce the effects low quality genome sequences on the models as described and evaluated previously [35]. Briefly, read depths at every position in the Wuhan-Hu-1 reference genome were computed across the larger set of genomes, and any position with an average depth lower than 10 (averaged across all genomes) was masked with an N character. This resulted in the masking of 56 internal positions in in every genome, which were not used in subsequent models. These included positions 22029–22033, 22340–22367, 22897, 22899–22905, and 23108–23122, which correspond with spike amino acid positions 156, 157, 260–269, 445–448, and 516–520. The low average coverage is likely the result of poor primer binding. The first and last 100 nucleotides in each genome were also masked to prevent jagged edges from creating variation that could be incorrectly learned by the models. Overall, this resulted in a total of 256 masked positions in each genome sequence. If any assembled genome had greater than 500 ambiguous characters in addition to this initial set of 256 masked positions, it was discarded.

### Feature matrix construction

In previous work, we showed that models built from assembled SARS-CoV-2 genomes could predict Ct values with modest predictive power [35]. In this study, we wanted to see if the addition of data from the raw reads, prior to the assembly step, could improve the Ct value predictions. To do this, we built models directly from the reference-based alignments (e.g., the pileup files), rather than the assembled sequences. The pileup file captures the counts of A,T,G,C, insertions, and deletions for each read aligning to a given position in the reference genome. A matrix was constructed where each row was a genome and each base position in the reference genome was represented as 6 columns: the counts of A,T,G,C, insertions, and deletions. This was followed by a one-hot encoding of the detection instrument.

Importantly, the modelling approach described below can incorrectly identify patterns in the data set that result from variations in read depth at each position, rather than true biological differences between samples, thus resulting in erroneously high R^2^ values. To prevent this, the count of each value [A,T,G,C, Insertion, Deletion] was divided by the sum of all values for a given alignment position. Each value was then assigned to the appropriate corresponding bin: [0–0.2), [0.2–0.4), [0.4–0.6), [0.6–0.8), or [0.8–1.0] to create the tuple for the position. In this way, the variations in depth could not create fractional values that could be read as signatures of a given Ct value.

### Model generation and evaluation

Unless otherwise stated, all models were generated using Extreme Gradient Boosting (XGBoost) [52] version 0.81 as a regression model for predicting Ct values from the pileup matrix. XGBoost regression was chosen based on a comparison of methods and hyper parameter tuning experiments, which were described previously [35]. Briefly, column and row subsampling were set by tree and at 75%, learning rate was set to 0.0625, and tree depth was set to 4. The models were evaluated using ten-fold cross validation with a train-test-split-evaluation split of 80% and 10%, and 10% of the data set respectively. For each fold, the R^2^ score and root mean square error (RMSE) were computed. Unless otherwise stated, data from the first five folds are shown.

Since it is difficult to conceptualize accuracy for a regression model, the model accuracy within Ct-value intervals of 6, 5, 4, 3, 2, and 1 was calculated. The accuracy within a given interval *n* was computed as the fraction of the samples where the absolute difference between the predicted and actual value was smaller than *n*.

### Predicting Ct values in new variants

To assess the model’s ability to predict the Ct values for a newly emerging variant, we varied the amount of Omicron sequences in the training set to see how well models built from previous variants could predict Omicron Ct values. A separate model was trained using a training set with the following percentages of Omicron genomes: 0%, 0.5%, 1%, 2%, 5%, 10%, and 15%. For each percentage, *n*, the corresponding number of Omicron genomes were added to the training set. The test set remained unchanged for all percentages across each fold. Models were evaluated against the testing sets and a held out set of Omicron genomes.

### Feature importance

In order to display regions of the Wuhan-Hu-1 genome where there are differences in iSNV frequencies relating to differences in the range of Ct values. We computed the frequencies of A,T,G,C, insertion, and deletion at each position relative to the reference genome. For a given position, if greater than ≥40% of the reads contained a given base, insertion, or deletion, then that genome contributed to the average displayed for that character at that position.

Feature importance was measured using the “gain” and “weight” metrics in XGBoost. Gain is a measurement of the reduction in error in the model due to the addition of a given feature. The weight is the number of times that feature is chosen by the trees in the model.

### External holdout data

In order to compare model results with other sequenced genomes, a data set of 1795 SARS-CoV-2 genomes and their corresponding Ct values were downloaded from SRA (**S4 Table in S1 File**). All genomes were processed and evaluated as described above.

## Supporting information

S1 Fig Dot plot depicting the XGBoost feature importance (weight). (PDF)

S1 File (S1) Samples used in this study, (S2) The distribution of samples and Ct values across instruments, (S3)R2 and RMSE values for outpatient, inpatient, IMU, and ICU samples, and (S4) Holdout set of external genomes with published Ct values from SRA.
(XLSX)

## Abbreviations

BV-BRC: Bacterial and Viral Bioinformatics Resource Center
Ct: cycle threshold
iSNV: intrahost single nucleotide variant
RMSE: root-mean-square error
RT-PCR: reverse transcription-polymerase chain reaction
VOC: variant of concern
XGBoost: extreme gradient boosting
