# Supplementary material for: Using intrahost single nucleotide variant data to predict SARS-CoV-2 detection cycle threshold values
Source: PLoS One. 2024 Oct 30;19(10):e0312686. doi: 10.1371/journal.pone.0312686 (PMC11524481; doi:10.1371/journal.pone.0312686)
Supplement: S1 Fig — (PDF) [file pone.0312686.s001.pdf]

Supporting Figures

Using intrahost single nucleotide variant data to predict SARS-CoV-2 detection cycle threshold values

Lea Duesterwald, Marcus Nguyen, Paul Christensen, S. Wesley Long, Randall J. Olsen, James M. Musser, and James J. Davis

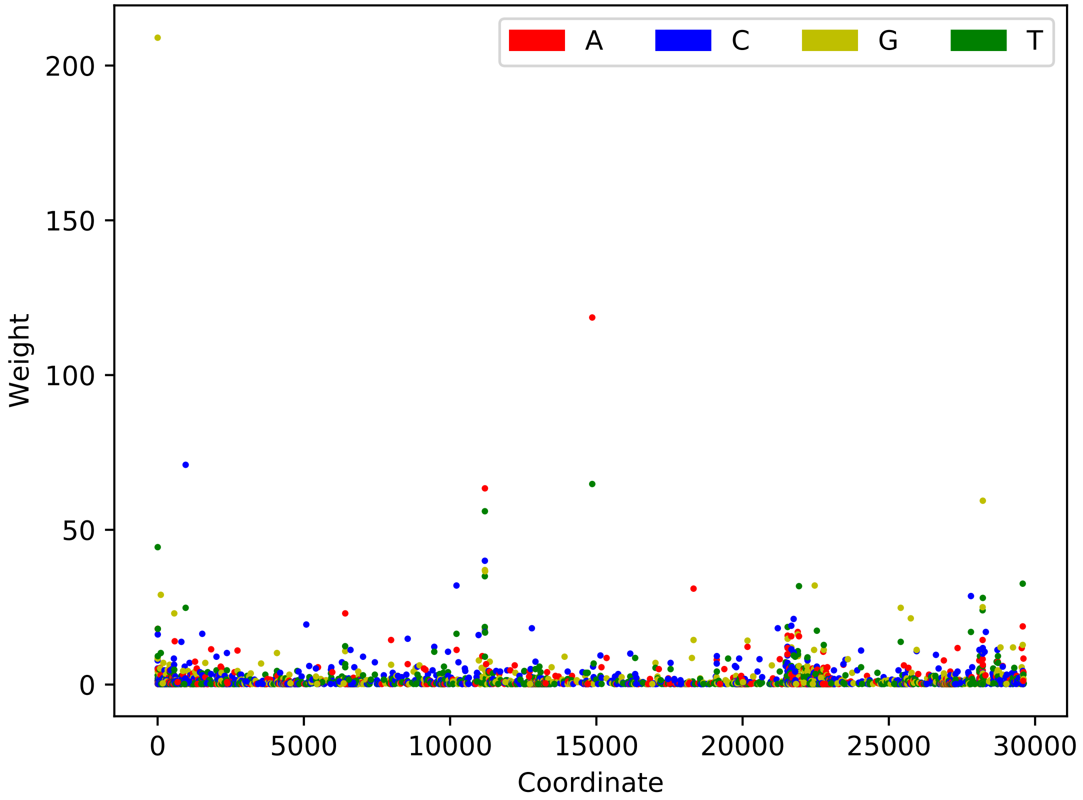

S1 Figure. Dot plot depicting the XGBoost feature importance (average weight) for each position and character used by the all-instrument model. Each base at a given position is colored according to the key. Genomic positions correspond to the SARS-CoV-2 Wuhan-Hu-1 reference genome. For each position, only genomes where ≥40% of the characters in the column corresponded to a given nucleotide were used to generate the average weight in order to reduce noise in the image. Additionally, only statistically significant bases are included, significance was computed based on the 95% confidence interval of the average Ct value of genomes with a given base and those without. No INDEL features met this significance requirement. The spike protein corresponds to genomic coordinates 21563–25384.
